# A new dataset for video-based cow behavior recognition

**DOI:** 10.1038/s41598-024-65953-x

**Published:** 2024-08-12

**Authors:** Kuo Li, Daoerji Fan, Huijuan Wu, Aruna Zhao

**Affiliations:** College of Electronic Information Engineering, Inner Mongolia University, College Road No. 235, Hohhot, 010021 Inner Mongolia Autonomous Region China

**Keywords:** Video sequence, Dairy cow behavior recognition, Dataset, SlowFast model, Information theory and computation, Electrical and electronic engineering

## Abstract

A new video based multi behavior dataset for cows, CBVD-5, is introduced in this paper. The dataset includes five cow behaviors: standing, lying down, foraging,rumination and drinking. The dataset comprises 107 cows from the entire barn, maintaining an 80% stocking density. Monitoring occurred over 96 h for these 20-month-old cows, considering varying light conditions and nighttime data to ensure standardization and inclusivity.The dataset consists of ranch monitoring footage collected by seven cameras, including 687 video segment samples and 206,100 image samples, covering five daily behaviors of cows. The data collection process entailed the deployment of cameras, hard drives, software, and servers for storage. Data annotation was conducted using the VIA web tool, leveraging the video expertise of pertinent professionals. The annotation coordinates and category labels of each individual cow in the image, as well as the generated configuration file, are also saved in the dataset. With this dataset,we propose a slowfast cow multi behavior recognition model based on video sequences as the baseline evaluation model. The experimental results show that the model can effectively learn corresponding category labels from the behavior type data of the dataset, with an error rate of 21.28% on the test set. In addition to cow behavior recognition, the dataset can also be used for cow target detection, and so on.The CBVD-5 dataset significantly influences dairy cow behavior recognition, advancing research, enriching data resources, standardizing datasets, enhancing dairy cow health and welfare monitoring, and fostering agricultural intelligence development. Additionally, it serves educational and training needs, supporting research and practical applications in related fields. The dataset will be made freely available to researchers world-wide.

## Introduction

Behavior recognition, harnessing the prowess of computer vision and related technologies, extracts and interprets behavioral cues from video data, playing a pivotal role in applications such as anomaly alerts, agricultural breeding, and animal behavior analytics. In the realm of dairy farming, automating the identification of cow behaviors within expansive pastures promises to augment bovine health, optimize resource allocation, and bolster the overall productivity of the livestock sector.

In the Inner Mongolia Autonomous Region, blessed with expansive grasslands and a favorable climate, dairy cow breeding plays a pivotal role, with the region serving as a major hub in China. According to the Regional Bureau of Statistics, by 2023, the dairy cow population escalated to 1.687 million, witnessing a 6.1% growth, accompanied by a milk production increase to 7.926 million tons, up 8.0%. Furthermore, large-scale dairy output surged to 4.73 million tons, representing a significant 13.2% annual increase^1^.

While the thriving dairy industry bolsters rural livelihoods and contributes to the transformation of agriculture, it grapples with challenges such as enhancing feed efficiency, managing diseases effectively, and advancing sustainability practices^2^. Amid these pressing issues, the importance of understanding cow behavior escalates, underscoring the necessity for comprehensive datasets to facilitate the development of efficient recognition models that can address these complexities.

A comprehensive dataset on cow behavior would enhance model effectiveness, improve farm management, boost productivity, and support sustainable livestock practices. By standardizing data collection, researchers can gather diverse, large-scale information covering feeding, resting, movement, and social behaviors.

To tackle the dearth of a high-quality dataset, a streamlined system integrating camera surveillance, data storage, custom software, and server infrastructure was deployed, leading to the development of a robust dataset. With expert dairy farmers’ insights, meticulous annotation refined the dataset, eliminating abnormalities to ensure purity and relevance for superior model training and livestock management enhancements. Establishing such a practical, all-encompassing database, rooted in real-ranch scenarios, is vital for progressing cow welfare, optimizing resource allocation, boosting dairy productivity, and fostering a global understanding of cow behavior among researchers.

Therefore, this study introduces a comprehensive cows behavior dataset named “CBVD-5”. This dataset is intended to serve as a benchmark for cow behavior recognition.The CBVD-5 dataset incorporates five atomic behavioral categories: standing and lying as posture-related behaviors, with foraging, rumination, and drinking classified as state behaviors. This taxonomy captures the intricate complexity of cow behavior, integrating both postural and state elements to accurately depict daily activities. Rigorously validated by empirical evidence gathered through close collaboration with dairy farmers, our classification schema ensures alignment with genuine farming scenarios, thereby enhancing its practical applicability in the field..

Categories were meticulously selected via dialogues with pasture proprietors and surveys of farmers to accurately reflect cows’ activity, rest, hydration, and feeding habits, collectively presenting a holistic view of their living standards and health. The dataset will be freely disseminated to the research community, facilitating advancements in cow behavior studies such as multi-animal detection, individual tracking, and multiplex behavior recognition, thereby stimulating innovations in this domain. The main contributions of this paper thus can be summarized as follows:

- A benchmark dataset, CBVD-5, is introduced in this paper for identifying cow behavior under standardized ranches. The dataset was collected using seven cameras, comprising 687 video segment samples and 206,100 image samples.The monitoring was conducted for 96 h on 20-month-old cows, taking into account different light conditions and nighttime data to ensure standardization and inclusiveness.
- An integral component of our work entails the development of specialized code tools and innovative device design strategies. These methodologies are pivotal for the streamlined collection, preprocessing, and meticulous cleansing of the cow behavior video dataset, thereby enhancing data quality, reducing noise, and optimizing the dataset’s utility for advanced research purposes.
- Lastly, we present the modification and adaptation of a benchmark recognition model leveraging the SlowFast architecture. Tailored specifically for recognizing and analyzing cow behaviors in standardized ranch settings, this adjusted model harnesses the temporal and spatial prowess of SlowFast to deliver enhanced accuracy in behavior recognition, thereby advancing the precision and applicability of livestock behavior analytics.

The remainder of the paper is organized as follows. “Related work” presents a literature review of Cows Behavior Videos Dataset. In “Overview of CBVD-5”, we present the data collection steps used in this study and the dataset statistics. “Benchmark evaluation” details the data preprocessing and framing process. Also it presents the adapted model and the experiment results of the cow behavior recognition algorithm using the CBVD-5 dataset. Lastly, we present the conclusions of this study in “Conclusion”. Finally, in “Prospects”, we also provide an outlook on and discuss extensions to our future research endeavors.

## Related work

Recent advancements in cow behavior recognition research can be broadly categorized into sensor-based methodologies and computer vision-based approaches. Sensor-based studies have utilized GPS positioning sensors for non/-intrusive monitoring of cow activities, achieving high classification accuracies through preprocessing and machine learning techniques^3^. Expanding upon this, Riaboff et al. combined accelerometer and GPS data to explore pasture behavior insights in dairy cows^4^. Sensor research has also ventured into multi-sensor and wearable devices, with Tian et al. achieving real-time recognition through geomagnetic and acceleration fusion models^5^, and Lovarelli et al. developing a sophisticated wearable sensor node for cow behavior classification^6^.

On the computational front, Li et al. integrated multiple strategies for comprehensive dairy cow behavior analysis^7^, while Guo et al. detected mounting behaviors via video analysis^8^, and Girish et al. focused on static image action recognition^9^. Avola et al. advanced 2D skeleton-based recognition with LSTM-RNNs^10^, showcasing the versatility and adaptability of computer vision for real-time monitoring and detailed behavioral analysis in various settings.

Sensor and vision-based studies have also seen applications in individual animal recognition, as demonstrated by Bhole et al.^11^, and health monitoring, such as lameness detection by Jiang et al.^12^, and respiratory behavior monitoring by Wu et al.^13^.Fuentes et al. developed a deep learning approach for recognizing hierarchical cattle behavior using spatio-temporal information^14^. Bai et al. introduced X3DFast, a 3D convolution-based model for efficient behavior classification^15^.

Despite these progresses, the availability of public datasets remains limited. Notable exceptions include COW-VID^16^ for calving prediction, COW-IMU^17^ for IMU data classification, and COW-Act^18^ for barn behavior tracking.

To address the scarcity of comprehensive datasets, we proudly introduce CBVD-5, featuring meticulous recordings of atomic behaviors: standing, lying, grazing, rumination, and drinking, all within a meticulously managed pasture environment. Rigorously validated, this standardized video dataset establishes a solid foundation for advancing our insights into cow behavior and improving livestock management practices.

## Overview of CBVD-5

### Cow behavior selection

The dataset utilized in this study serves as a foundational resource for research and experimentation with deep learning algorithms. Over the years, researchers have proposed various human behavior datasets such as HMDB-51, UCF-101^19^, and Kinetics-400^20^, leading to the development of numerous novel behavior recognition algorithms.

However, there is currently no publicly available video behavior dataset specifically tailored to cows under standardized ranch conditions. Therefore, the primary objective of this study is to construct a comprehensive cow behavior dataset. To determine the specific behaviors to include, extensive investigations, evidence collection, and consultations with ranch owners were conducted. As a result, five key behaviors were identified: standing, lying down, foraging, drinking water, and rumination.

In our research, we adopt a systematic classification approach, defining the five fundamental behaviors as elemental behaviors: standing and lying down are labeled as “postural behaviors,” representing the basic stances cows adopt. Meanwhile, the actions of foraging, drinking, and rumination fall under the category of “state behaviors,” elucidating the active processes or physiological conditions they undergo at any given time. This framework serves to decode more complex behavioral patterns by focusing on their essential components.

Observing a cow’s standing behavior helps understand its activity patterns and vitality; metrics such as duration and recurrence frequency indicate comfort and exercise adequacy. Lying down, a pivotal rest period, rejuvenates energy, with close monitoring of rest intervals contributing to wellness assessments. Analyzing foraging behavior, central to nutrition and health, requires careful examination for assessing dietary adoption efficiency and digestive health, achieved by observing the frequency, duration, and intervals of foraging activities. Similarly, scrutinizing drinking patterns offers insights into hydration levels and water preferences, critical for overall health maintenance. Furthermore, evaluating rumination, a foundation of digestive health, through assessing its frequency, duration, and patterns, highlights the suitability of feeding conditions, including aspects like forage quality, timing, and frequency. Collectively, meticulously tracking these behaviors provides a thorough understanding of the cows’ health, welfare, and productivity.

Table 1 provides a video-level description and understanding of the five behaviors exhibited by cows. These behaviors were carefully chosen to be comprehensive and representative. Studying and identifying these behaviors can optimize feeding management strategies, enable timely measures to reduce disease occurrence and spread, and facilitate prompt adjustments to feeding management practices, ensuring the health and productivity of cows. Furthermore, this dataset enables in-depth research on behavior patterns, individual differences, and group dynamics of cows. It aids in monitoring their activity levels, rest conditions, dietary intake, and digestive functions, thereby facilitating the timely identification of health issues, optimization of feeding management, and improvement of production performance and welfare levels. Ultimately, this dataset supports decision-making processes for relevant personnel, leading to enhanced cow health and production efficiency.

**Table 1:** Detailed Description of cow behaviors captured in CBVD-5 dataset.

Action category	Describe
Stand	Assuming a quadrupedal stance, with the body in an upright position and the head slightly elevated
Lying down	Assume a lateral recumbent position, with the body relaxed on the ground and the limbs extended to one side. They may also gently wag their tail from time to time
Foraging	Assume a quadrupedal stance, with their body upright and the head slightly elevated. Keep the mouth close to the trough and use their tongue to pull the food into their mouth. Chew thoroughly and swallow
Drinking water	Assume a position near the water source and either lap up the water with their tongue or drink directly with their head down. Keep their mouth open and closed as needed to drink comfortably
Rumination	Assuming a quadrupedal stance with the body in an upright position and the head slightly elevated, the jaw will move up and down in a regular pattern. They stares in a certain direction and swallows ruminate food

### Data collection

**Figure 1:**
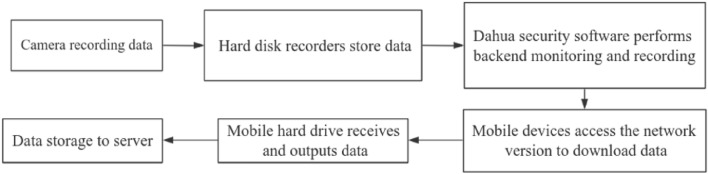
Architecture diagram of camera data storage and acquisition system.

Due to the increasing demand for surveillance and security, we have made the decision to utilize surveillance cameras for the purpose of collecting videos capturing cow behavior. By installing cameras on ranches, we can facilitate real-time monitoring and effective livestock management. The experimental equipment includes 2.8 mm and 3.6 mm Dahua cameras (M/K models), Dahua DH-S3000C-16GT 16-port gigabit network switches, and Dahua DH-NVR2216-HDS3 network hard disk recorders. For broader coverage areas, we have selected 2.8 mm lenses, while for monitoring distant targets, we have opted for 3.6 mm lenses. The layout plan for camera installation on the ranch, as well as the data storage and acquisition plan, have been designed and finalized. The overall system architecture is illustrated in Fig. 1.

The monitoring system’s installation and data acquisition commence with demarcating the surveillance zone, comprising feeding, watering, and other key areas. Fixed infrared cameras are strategically selected to enable uninterrupted 24/7 video monitoring and recording. Positioned approximately every 10 m with a 17$\circ$ downward tilt, these cameras, mounted securely on walls to avoid obstructions, ensure comprehensive coverage of the targeted zones.

To connect the cameras to the network, a wired connection is selected, utilizing Power over Ethernet (PoE) for power supply. A hard disk recorder is chosen as the storage device for storing the surveillance footage. The cameras are connected to the existing distribution box in the ranch to ensure a stable power supply. Additionally, auxiliary facilities such as distribution boxes, clamps, network cables, and network switches are installed as necessary to enhance monitoring effectiveness and security.

**Figure 2:**
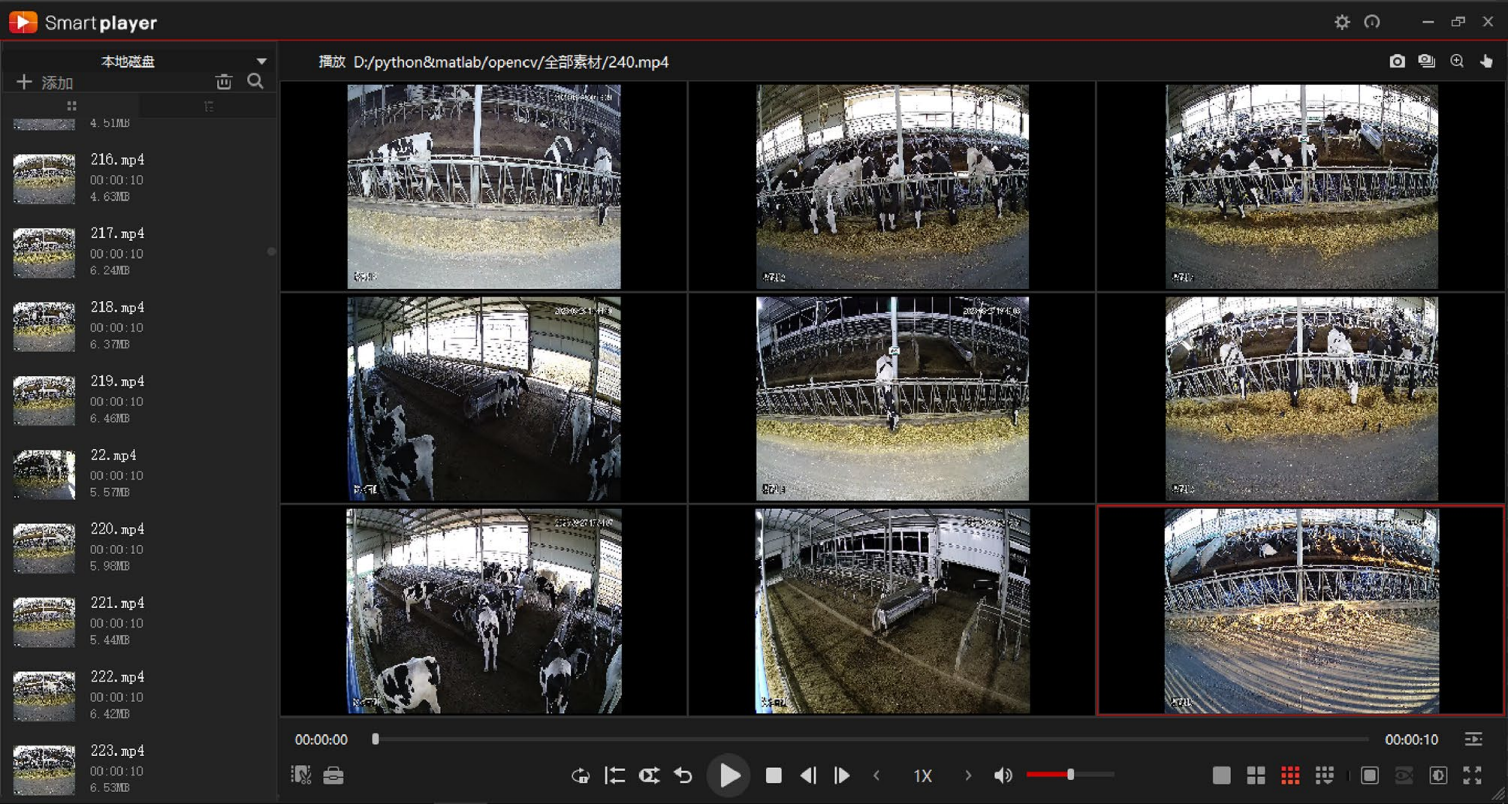
Main page of Dahua player.

Video data from surveillance is integrated into the Dahua Security Software (depicted in Fig. 2), enabling remote access via its web interface on mobile devices. Subsequently, data is extracted onto a portable hard drive and relayed to the central server, thus concluding the data gathering phase. Fundamentally, the system is tasked with dual operations: continuous data acquisition and routine monitoring.

### Data annotations

After collecting the video data, to reduce the workload of manual annotation, the decision was made to extract frames and select keyframes for manual annotation. The annotation tool chosen was via 3.0.11, and the annotation format followed that of the ava dataset. A total of 206,100 image data were generated from the frame extraction process, out of which 4122 keyframes were selected for annotation. The annotations included checkboxes for selecting targets and category labels. To minimize labeling errors, the images were annotated in batches by our researchers, and the labeled data was later reviewed and improved by experienced personnel. As a result, we obtained a total of 27,501 valid labeled data.

### Dataset statistics

Now, the CBVD-5 dataset is publicly available at https://www.kaggle.com/datasets/fandaoerji/cbvd-5cow-behavior-video-dataset to all researchers.

**Figure 3:**
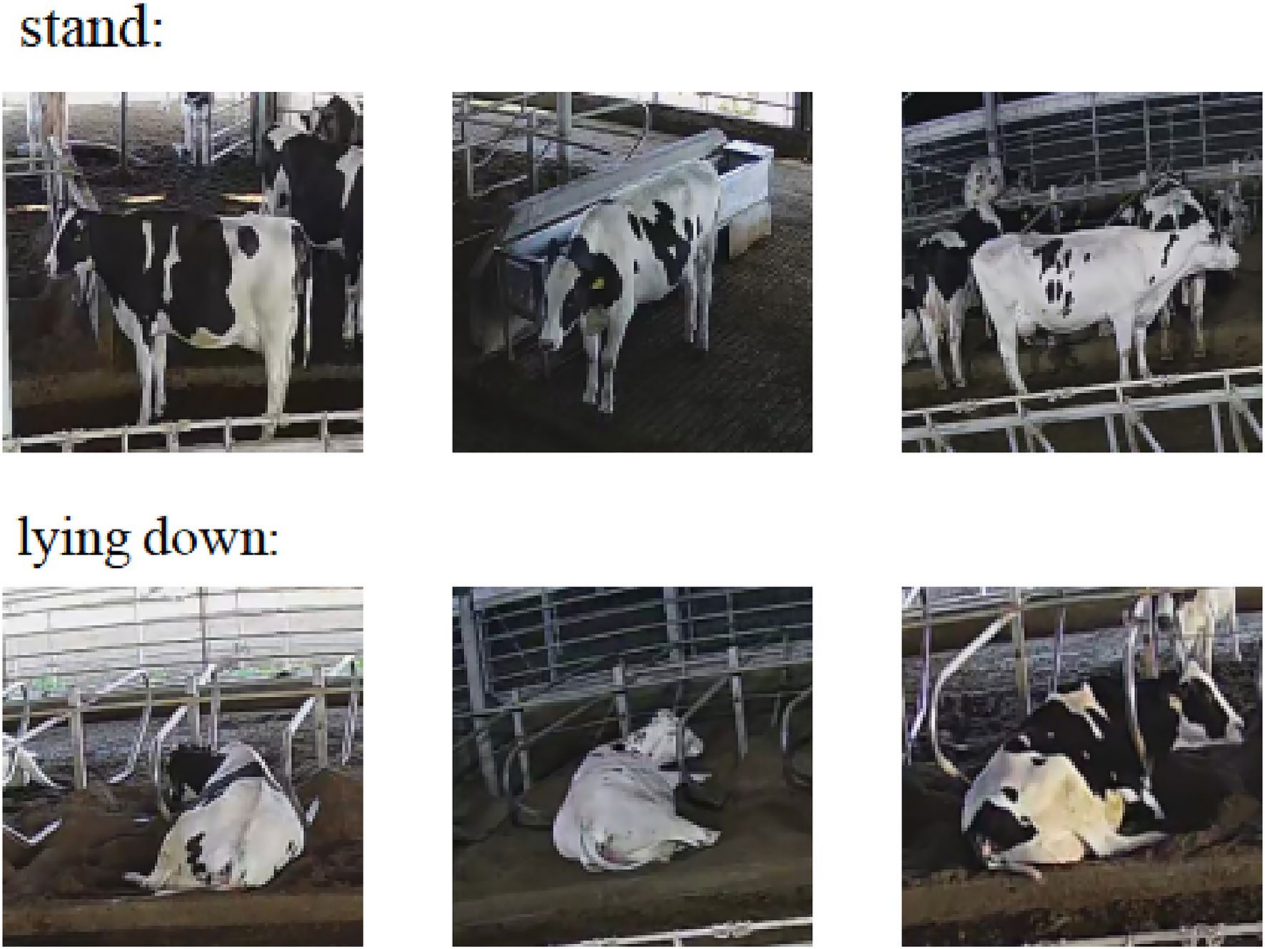
Two postural behavior samples in CBVD-5.

**Figure 4:**
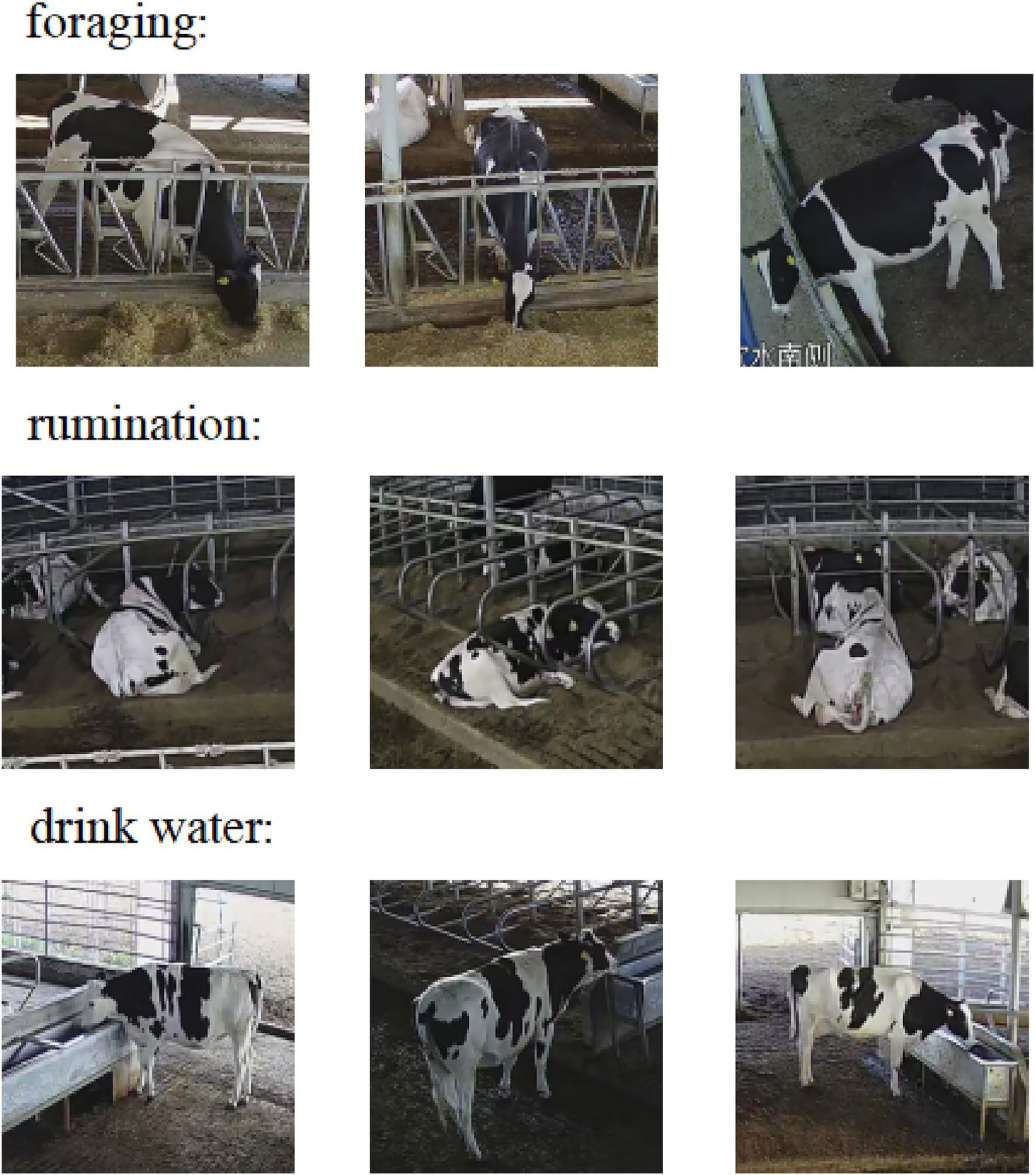
Three state behavioral samples in CBVD-5.

The dataset encompasses labeled data, keyframe images, and raw video footage procured via camera installations, as illustrated in Fig. 3. These keyframe images depict two categories under the umbrella of “postural behaviors”: standing and lying down. Further, Fig. 4 exhibits keyframe illustrations for three classifications categorized as “state behaviors” within the dataset: foraging, rumination, and drinking.The CBVD-5 dataset contains a total of 206,100 image samples, and no separation of training and test sets. Users can divide the test set and training set according to their own needs.

**Figure 5:**
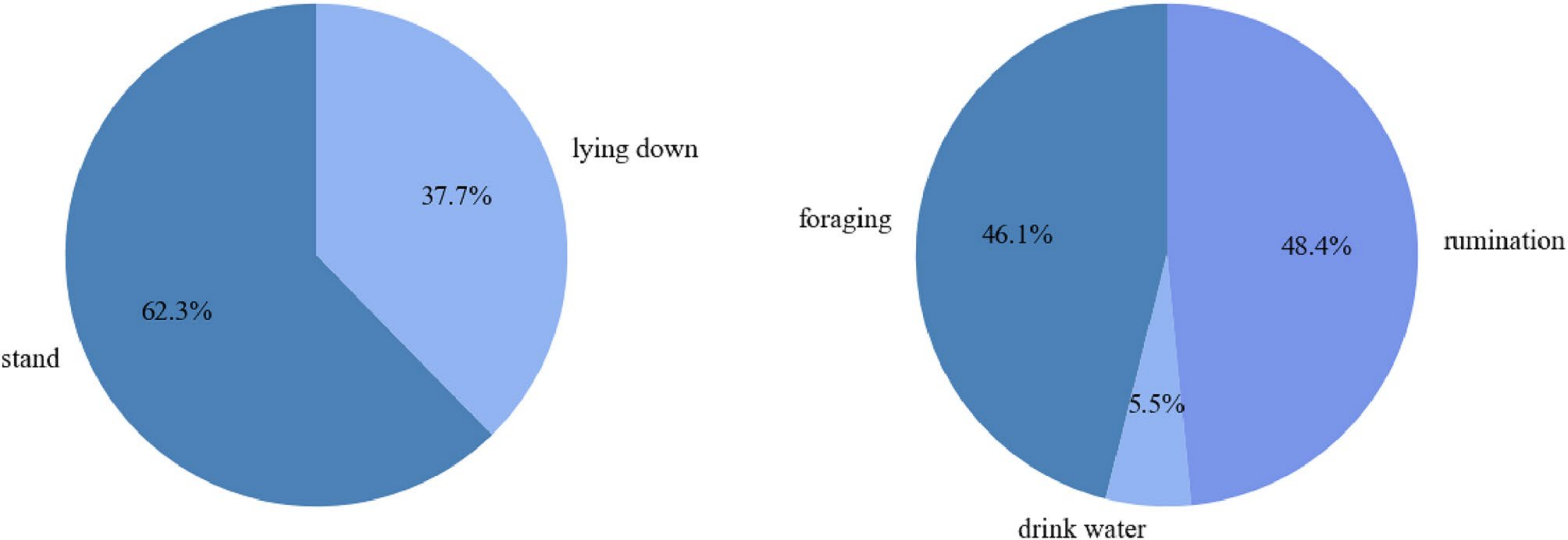
Proportional composition of five categorized behavioral samples.

We performed a discrete statistical analysis on the count of all “atomic behavior” categories within the dataset. As illustrated in Fig. 5, the diagram furnishes an overview of the compositional proportions of both “postural behaviors” and “state behaviors,” offering insights into their respective representations within the dataset.

**Figure 6:**
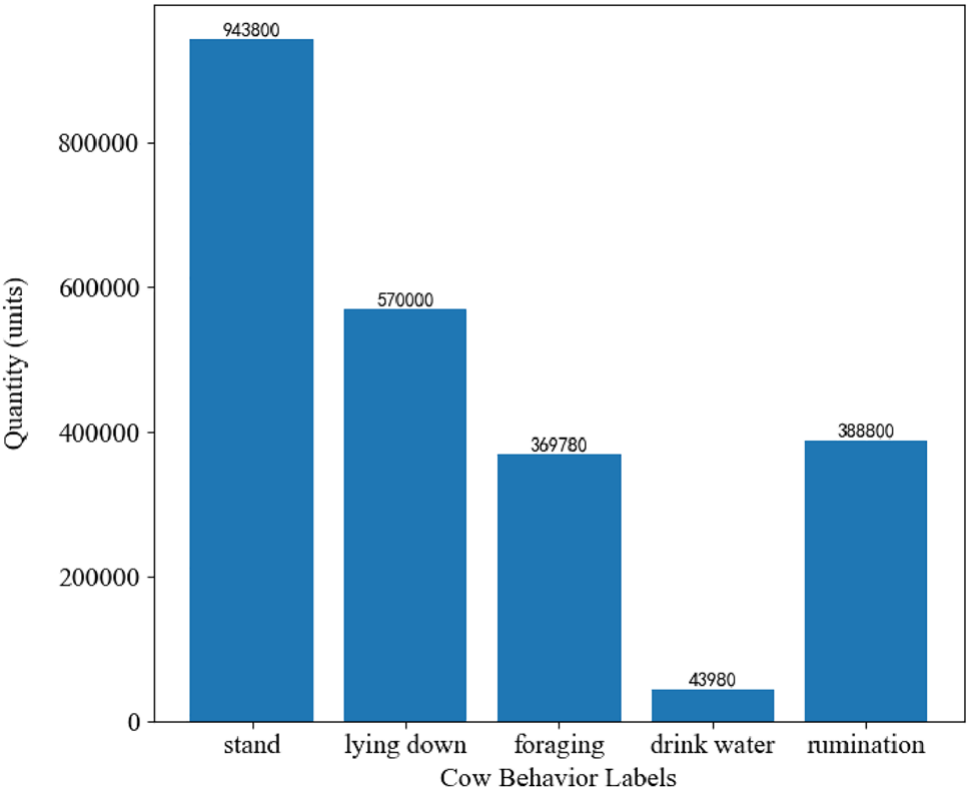
Sample count statistics for five classes of atomic behaviors.

The dataset comprises 206,100 image samples, and a detailed presentation of the distribution of these samples based on category labels from the annotation files is depicted in Fig. 6,which shows the number of occurrences of each atomic behavior in the sample files.

It is noteworthy that the CBVD-5 dataset contains a significant number of keyframe image samples labeled as “lying down,” indicating that lying down is a common atomic behavior among cows in the dataset.Furthermore, the dataset encompasses diverse combinations of both postural and state behaviors within the realm of atomic actions, including illustrative pairings such as standing coupled with drinking and lying down accompanied by rumination, among others. This comprehensive inclusion fosters a deeper understanding of the intricate interplay between these fundamental behaviors in cows.

**Figure 7:**
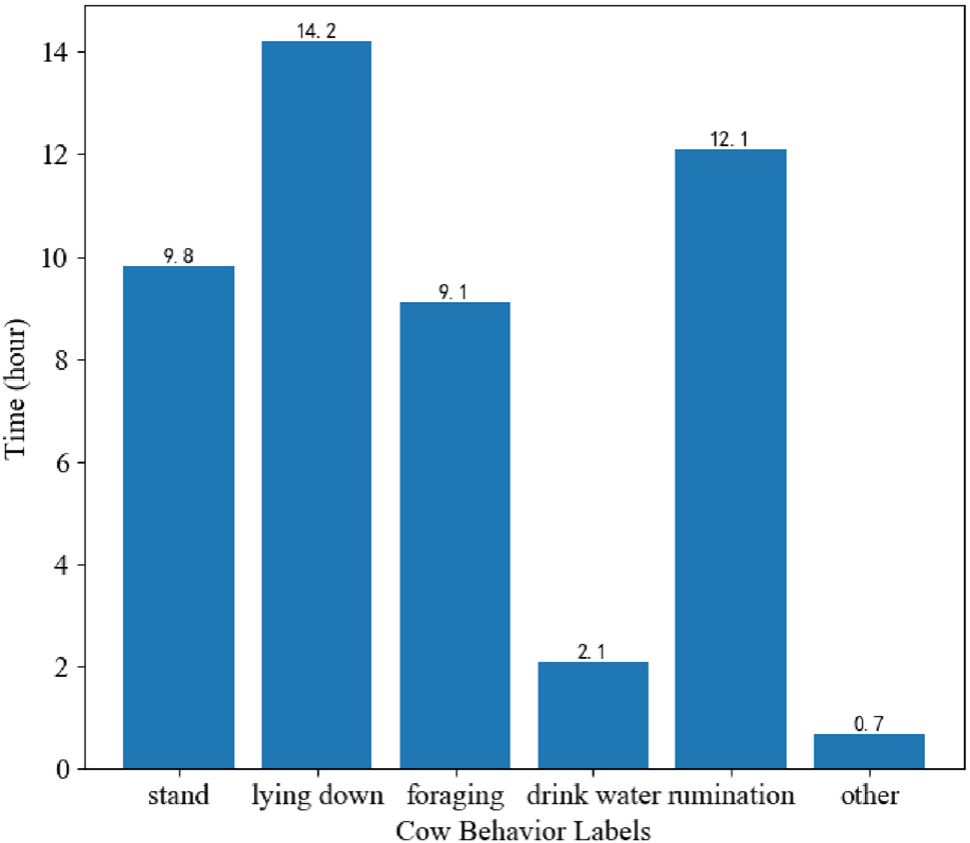
Statistical chart of daily cow behaviors.

In addition, our research team has validated and analyzed the behavioral patterns and regularities of cows in the pasture where the data set was collected over a 24-hour period. We have represented these patterns using a bar chart, as shown in Fig. 7. By comparing and analyzing the composition of our CVBD-5 dataset, we have found that the proportions of different behavioral categories in the CBVD-5 dataset are scientifically grounded.

The dataset consists of five folders, namely “videos”, “video_cut”, and “rawframes”,“Labelframes”, “annotations”. Among them, videos represent the original video, and video_cut is the cropped 10s video segment sample.Rawframes are images generated by video sample frame segmentation for training, while labelframes are generated images for keyframe extraction for annotation.Annotations contains 8 files, which are all annotation files generated by annotations. Among them, there are 5 types of atomic behaviors in “ava-action_listv2.1.pbtxt.txt”, among which the most important is the. csv file for annotating the data. Taking “avatrain_v2.2. csv” as an example, it is the training dataset file, where the key information includes 5 parts: [Video_id, middle_Frame_timestamp, Object_detection_box, Action_ID, Target_ID]

1. Video_ID: Video Name
2. Middle Frame Timestamp: The position of the keyframe (in seconds)
3. Object_detection_box: This includes four columns, (x1, y1, x2, y2), which represent the positions of the upper left and lower right points after normalization of the original labeled data.
4. Action_Id: ava Action List The corresponding IDs in v2.1. pbtxt are for five category labels.
5. Target_ID: Animal category: cow, so its default value is 1. Each row is labeled with only one box, one task, and corresponding multiple category labels.

“ava_train_excluded_timesampsv2.1.csv” is the frame in which the problem occurred in the evaluated dataset, used for validation “ava_dense_Proposals_train.FAIR.recall_93.9.pkl” and “ava_dense_Proposals_val.FAIR recall_93.9.pkl” are used to cache data and reduce the computation time of Python programs for subsequent processing.

## Benchmark evaluation

Cow behavior recognition can be seen as a process combining object detection and behavior classification. As a pilot recognition task, we chose to use the SlowFast model from the mmaction2 toolkit to complete this task.

Before selecting the SlowFast model, we thoroughly investigated the shortcomings of other behavior recognition models in handling datasets like AVA. While 2D CNNs excel at extracting spatial features from video frames, they lack the ability to capture temporal information, limiting their efficacy in dynamic behavior analysis^21^. 3D CNNs directly process spatiotemporal information from video sequences, but their high computational cost and training difficulties hinder their efficiency, particularly when dealing with lengthy video sequences^22^.Although C3D models have achieved some success, their performance diminishes when confronted with long sequence videos, struggling to effectively capture intricate details and long-term dependencies^23^.

The SlowFast model analyzes a sequence of images based on the preceding and succeeding frames to determine the performed action. For behavior recognition, where actions vary while the environment remains constant, the idea behind the SlowFast model is to separately extract action information and environmental information, and then fuse them together to accomplish behavior recognition.

### Data of video preprocessing

After completing the data collection, the video data needs to undergo preprocessing.Referring to the impressive performance of SlowFast on AVA data, we aim to apply transfer learning to cows and construct a dataset similar to AVA data types.

Firstly, we need to preprocess the video data. The original surveillance videos are encoded in H.265/HEVC format. To facilitate subsequent processing, we will utilize the Moviepy library to convert them to H.264/AVC encoding.Next, we will select data segments from the videos. The original surveillance videos generate a recording every hour, resulting in a total of 700 videos from all cameras with a combined duration of 42,000 min. To ensure randomness, we will create the dataset by randomly selecting a starting time point within a long video segment. We will then capture a fixed duration of 10 s of video segments. We will exclude the segments before IP allocation, resulting in a remaining count of 687 video segments.Finally, since the original SlowFast model requires video processing with 30 frames and a frame rate of 25 frames per second, we will utilize the ffmpeg tool to increase the frame rate of the video segments. This step ensures that the video segments meet the requirements for subsequent processing and generation tasks.

Preprocessing and framing:

1. Convert the original surveillance videos from H.265/HEVC format to H.264/AVC encoding for subsequent processing.
2. Randomly select a starting time point to create a dataset from long video segments.
3. Capture fixed-duration video segments of 10 seconds.
4. Exclude segments before IP allocation to ensure dataset quality and accuracy.
5. Use the ffmpeg tool to increase the frame rate of video segments to meet the requirements of subsequent processing and generation tasks.
6. To comply with the requirements of the SlowFast model, ensure that the frame rate of the video segments is increased to 30 frames per second using video processing techniques.

These steps lay the foundation for subsequent transfer learning and building a cow dataset similar to the AVA data type.

### Details of the proposed model

**Figure 8:**
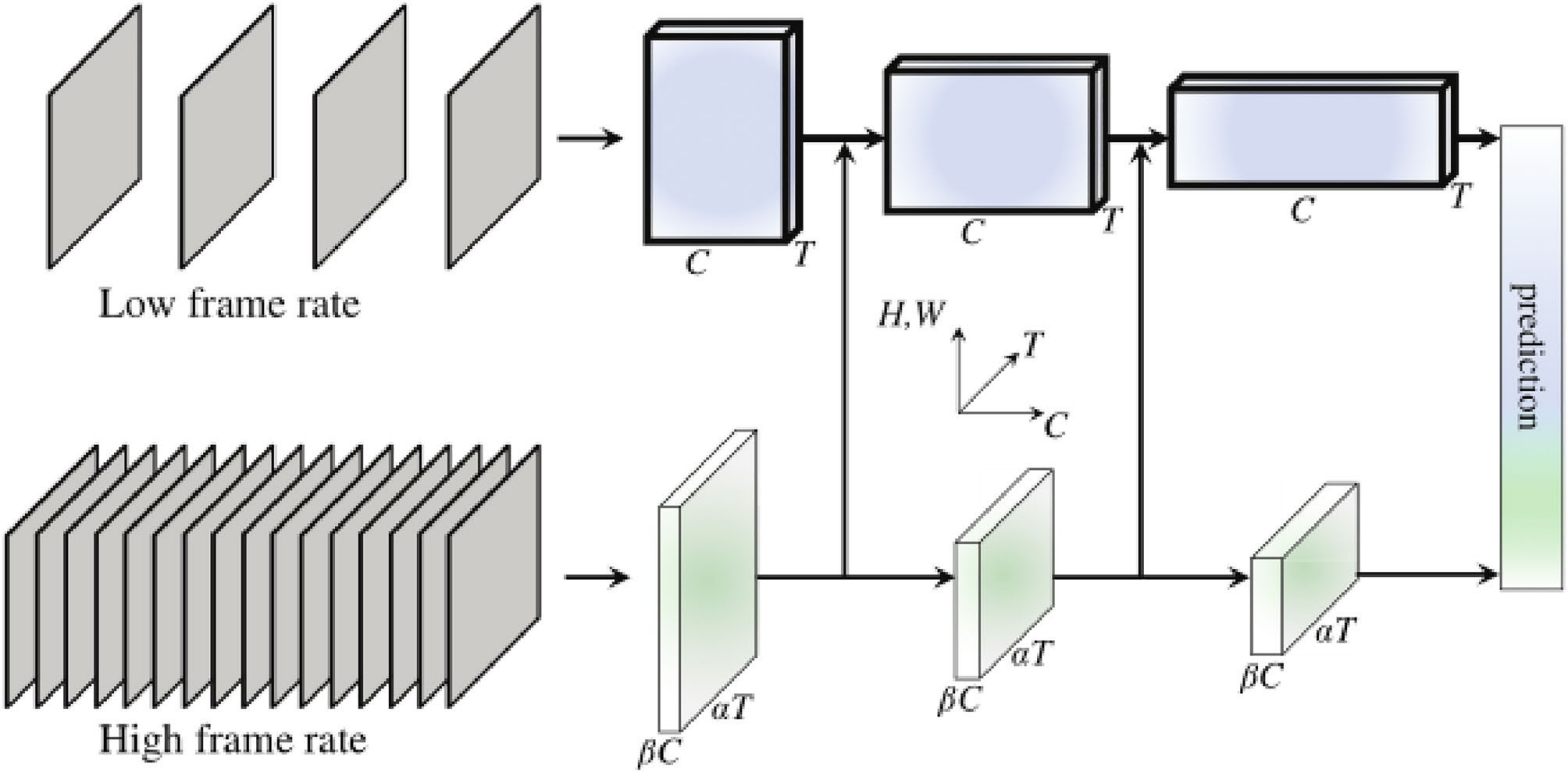
A SlowFast network.

The SlowFast model takes a video as input and undergoes feature extraction through the Slow Branch and Fast Branch. The Slow Branch processes the temporal information of the video by capturing long-term dynamics with a lower frame rate. The Fast Branch handles the spatial information of the video by capturing short-term dynamics with a higher frame rate^24^. The features extracted from the two branches represent temporal and spatial features, respectively.Next, the Slow Feature Fusion and Fast Feature Fusion modules merge the temporal and spatial features to integrate the two types of information. This fusion process can be achieved in various ways, such as merging feature maps or feature vectors.Finally, the fused features are fed into a classifier or regressor for the final classification or regression prediction. The output is the predicted result for the input video.As shown in the Fig. 8, this is the network architecture of SlowFast. The design of the SlowFast model enables it to simultaneously process the temporal and spatial information of videos, leading to a better understanding of video content and dynamic changes. It has achieved excellent performance in tasks such as video classification and action recognition.

In Table 2 is a detailed design of SlowFast based on ResNet50 as the backbone network. It can be observed that the frame rate of Fast is 8 times that of Slow (lightgreen), while the channel number of Slow is 8 times that of Fast (orange). Fast always maintains a relatively low channel capacity for lightweight consideration (faster), and Fast focuses more on temporal information, so it can ignore some spatial information.

**Table 2:** An example instantiation of the SlowFast network.

Stage	Slow pathway	Fast pathway	Output sizes T$\times S^2$
Raw clip	–	–	644$\times 224^2$
Data layer	Stride 16, $1^2$	Stride **2**, $1^2$	Slow: 4$\times 224^2$
			Fast: **32**$\times 224^2$
conv$_1$	1$\times 7^2$, 64	5$\times 7^2$, **8**	Slow: 4$\times 112^2$
	Stride 1, $2^2$	Stride 1, $2^2$	Fast: **32**$\times 112^2$
pool$_1$	1$\times 3^2$, max	1$\times 3^2$, max	Slow: 4$\times 56^2$
	Stride 1, $2^2$	Stride 1, $2^2$	Fast: **32**$\times 56^2$
res$_2$	$$\left[ \begin{array}{c} 1\times 1^2, 64\\ 1\times 3^2, 64\\ 1\times 1^2, 256 \end{array}\right] \times 3$$	$$\left[ \begin{array}{ccc} 3\times 1^2, {\textbf {{8}}}\\ 1\times 3^2, {\textbf {{8}}}\\ 1\times 1^2, {\textbf {{32}}} \end{array}\right] \times 3$$	Slow: 4$\times 56^2$
			Fast: **32**$\times 56^2$
res$_3$	$$\left[ \begin{array}{c} 1\times 1^2, 128\\ 1\times 3^2, 128\\ 1\times 1^2, 512 \end{array}\right] \times 4$$	$$\left[ \begin{array}{ccc} 3\times 1^2, {\textbf {{16}}}\\ 1\times 3^2, {\textbf {{16}}}\\ 1\times 1^2, {\textbf {{64}}} \end{array}\right] \times 4$$	Slow: 4$\times 28^2$
			Fast: **32**$\times 28^2$
res$_4$	$$\left[ \begin{array}{c} 3\times 1^2, 256\\ 1\times 3^2, 256\\ 1\times 1^2, 1024 \end{array}\right] \times 6$$	$$\left[ \begin{array}{ccc} 3\times 1^2, {\textbf {{32}}}\\ 1\times 3^2, {\textbf {{32}}}\\ 1\times 1^2, {\textbf {{128}}}\end{array}\right] \times 6$$	Slow: 4$\times 14^2$
			Fast: **32**$\times 14^2$
res$_5$	$$\left[ \begin{array}{c} 3\times 1^2, 512\\ 1\times 3^2, 512\\ 1\times 1^2, 2048 \end{array}\right] \times 3$$	$$\left[ \begin{array}{ccc} 3\times 1^2, {\textbf {{64}}}\\ 1\times 3^2, {\textbf {{64}}}\\ 1\times 1^2, {\textbf {{256}}}\end{array}\right] \times 3$$	Slow: 4$\times 7^2$
			Fast: **32**$\times 7^2$

Significant values are in bold.

SlowFast Networks use the Slow pathway to extract spatial semantic features (such as color, size, shape, etc.) from sparse RGB frames, and the Fast pathway to capture motion information (extracting temporal features) while reducing the number of channels to make it lightweight. The direct connection from Fast to Slow can fuse finer-grained spatiotemporal information at an early stage.

In the SlowFast model, the steps for object detection and recognition using Faster R-CNN and ResNet-50 are as follows:

1. Data preprocessing: resize, crop, and normalize images, and convert them into the required tensor format.
2. Feature extraction: use ResNet-50 to extract high-level features from the images.
3. Region proposal network (RPN): generate candidate object regions on the feature map and filter high-probability candidates.
4. Region of interest pooling (RoIPool): map selected candidate bounding boxes to a fixed-size feature map.
5. Object classification and bounding box regression: classify objects and refine their bounding box positions.
6. Non-maximum suppression (NMS): filter out duplicate detections based on confidence scores and overlapping areas.
7. Object recognition: optionally perform further recognition or attribute classification on the detected objects.

Our subsequent experiments on data set evaluation are conducted based on this network structure and model, with further adjustments of hyperparameters to complete the subsequent experiments.

### Experiment results

From preprocessed CBVD-5 dataset, we randomly selected 70% as the training set, 20% as the test set and the remaining 10% as the validation set. The cross-entropy loss function is used during training, and the training is stopped when the loss on the validation set is no longer reduced, and the epoch with the smallest loss on the validation set is selected as the optimal model. Each training epoch took nearly 160 s on the GPU NVIDIA Quadro P5000.

The SlowFast model has demonstrated impressive accuracy in the field of behavior recognition. For cow behavior recognition, we placed greater emphasis on adjusting hyperparameters such as the initial factor, adjustment factor, and learning rate in the training configuration file. The configuration file utilizes the parameter scheduler “param_scheduler” and the optimizer wrapper “optim_wrapper”. Initially, we employed a “LinearLR” type parameter scheduler to linearly adjust the learning rate based on the initial factor. Next, we used a “MultiStepLR” type parameter scheduler to adjust the learning rate based on the adjustment factor between the total training epochs according to the specified interval. Finally, we used an optimizer of type “SGD” to determine the learning rate, momentum, and weight decay. Additionally, we set the configuration for gradient clipping to ensure more stable model convergence during training.

During adjustment, a ResNet3D network with a depth of 5 and lateral connections was used for the slow pathway. The kernel size of the first convolutional layer was (1, 7, 7), and the spatial stride was (1, 1, 1, 1). The first pooling layer had a stride of 1 and a spatial stride of (1, 2, 2, 1). The fast pathway also used a ResNet3D network with a depth of 50, without lateral connections, and 8 channels. The kernel size of the first convolutional layer was (5, 7, 7), and the first pooling layer had a stride of 1 and a spatial stride of (1, 2, 2, 1).

After conducting our experiments, we found that the recognition accuracy for standing, lying, and feeding behaviors was relatively high, while rumination behavior was more difficult to recognize at the video level. Therefore, we selected the accuracy of rumination behavior as the evaluation metric. We chose mAP (mean Average Precision) and rAP (rumination Average Precision) as the evaluation metrics for the five types of behaviors, where rAP refers to the accuracy of rumination behavior.

The mean Average Precision (mAP) comprehensively evaluates a model’s accuracy and recall across different categories by computing the average precision, providing a comprehensive assessment of the model’s recognition accuracy across diverse behavior categories in multi-class behavior recognition tasks.

Moreover, the use of the rumination Average Precision (rAP) as an evaluation metric allows for a more focused assessment of the model’s performance specifically on rumination behavior recognition, which is crucial for reflecting the health of cows. The rumination Average Precision facilitates a nuanced understanding of the model’s accuracy in this specific behavior and can be utilized to compare the relative performance of different model structures or algorithms in rumination behavior recognition.

We conducted experiments to determine the optimal initial factor(start_factor), using only the initial factor as the independent variable. The results, shown in Table 3,indicate that the model performs best on the test set when the initial factor is set to 0.2. Therefore, all subsequent experiments were conducted with an initial factor of 0.2.

**Table 3:** Start factor tuning experiment result.

Start_factor	Train		Test	
	mAP (%)	rAP (%)	mAP (%)	rAP (%)
0.1	0.7592	0.6450	0.6490	0.3174
0.2	0.8532	0.7504	**0.7435**	**0.4074**
0.3	0.8681	0.6450	0.7130	0.3326
0.5	0.7610	0.6529	0.6936	0.3066

Significant values are in bold.

The second hyperparameter to be tuned is the adjustment factor gamma. The adjustment factor gamma is used to intervene in the specified training interval based on the adjustment factor. When tuning, other hyperparameters are selected empirically, and gamma is selected around 0.2. The experimental results are shown in Table 4, which demonstrate that the best performance is achieved when the adjustment factor gamma is set to 0.1.

Next, we fine-tuned the learning rate and compared the results when it was set to 0.1, 0.2, 0.5, and 0.8. The results, shown in Table 5, indicate that the model achieved the highest accuracy in behavior recognition when the learning rate was set to 0.5. Furthermore, we conducted an extensive comparison of the impact of using ResNet50 and ResNet18 in the slow and fast pathways on the accuracy of behavior recognition. We found that the impact on accuracy was negligible.

**Table 4:** Adjustment factor tuning experiment result.

Adjustment_factor	Train		Test	
	mAP (%)	rAP (%)	mAP (%)	rAP (%)
0.05	0.8266	0.7007	0.7075	0.3716
0.10	0.8713	0.7872	**0.7198**	**0.4744**
0.15	0.8285	0.7105	0.7112	0.4502
0.20	0.7138	0.6033	0.6626	0.1897

Significant values are in bold.

**Table 5:** Learning rate tuning experiment result.

Learning rate	Train		Test	
	mAP (%)	rAP (%)	mAP (%)	rAP (%)
0.1	0.8137	0.7088	0.6542	0.2026
0.2	0.8285	0.7105	0.6936	0.3497
0.5	0.8361	0.7326	**0.7120**	**0.5237**
0.8	0.7808	0.6773	0.6895	0.2193

Significant values are in bold.

Ultimately, by fine-tuning the SlowFast network structure with the appropriate hyperparameters, we achieved the best performance.

So far, we have determined the optimal values of three hyperparameters in the model training process: initial factor, adjustment factor, and learning rate, which are 0.2, 0.1, and 0.5, respectively. In addition, we also attempted to replace the model with ResNet18 and ResNet50, but found that they did not significantly affect the accuracy of behavior recognition, although they did increase training time. Currently, the accuracy of each behavior on the training and testing sets is shown in Table 6. The final accuracy achieved for standing, lying down, and eating was good, while accuracy for drinking and rumination was relatively low, indicating that further research is needed.

To yield these experimental results and analyze their implications, it’s evident that the model’s training process is skewed towards behaviors with a higher volume due to data imbalance, resulting in subpar recognition performance for less frequent behaviors. Furthermore, the similarity between behaviors confounds the model, leading to a decrease in recognition accuracy.

To enhance the model’s efficacy, future endeavors could entail data augmentation techniques, such as rotation, scaling, and cropping, to diversify the dataset, aiding the model in discerning between various behaviors. Addressing the data imbalance issue could involve employing resampling techniques to balance sample quantities or utilizing a weighted loss function to prioritize behaviors with fewer instances, thereby increasing the model’s focus on these behaviors. Additionally, enriching feature representations could assist the model in distinguishing between behaviors more effectively. Exploring more intricate model architectures or optimization algorithms, such as deep neural networks, transfer learning, or ensemble learning, could also be pursued to bolster the model’s performance and generalizability.

**Table 6:** Best experimental results for each behavior category.

Label	Train AP (%)	Test AP (%)
Stand	0.9970	0.9861
Lying down	0.9928	0.9671
Foraging	0.9134	0.9695
Drinking water	0.6660	0.3482
Rumination	0.7872	0.4100

**Figure 9:**
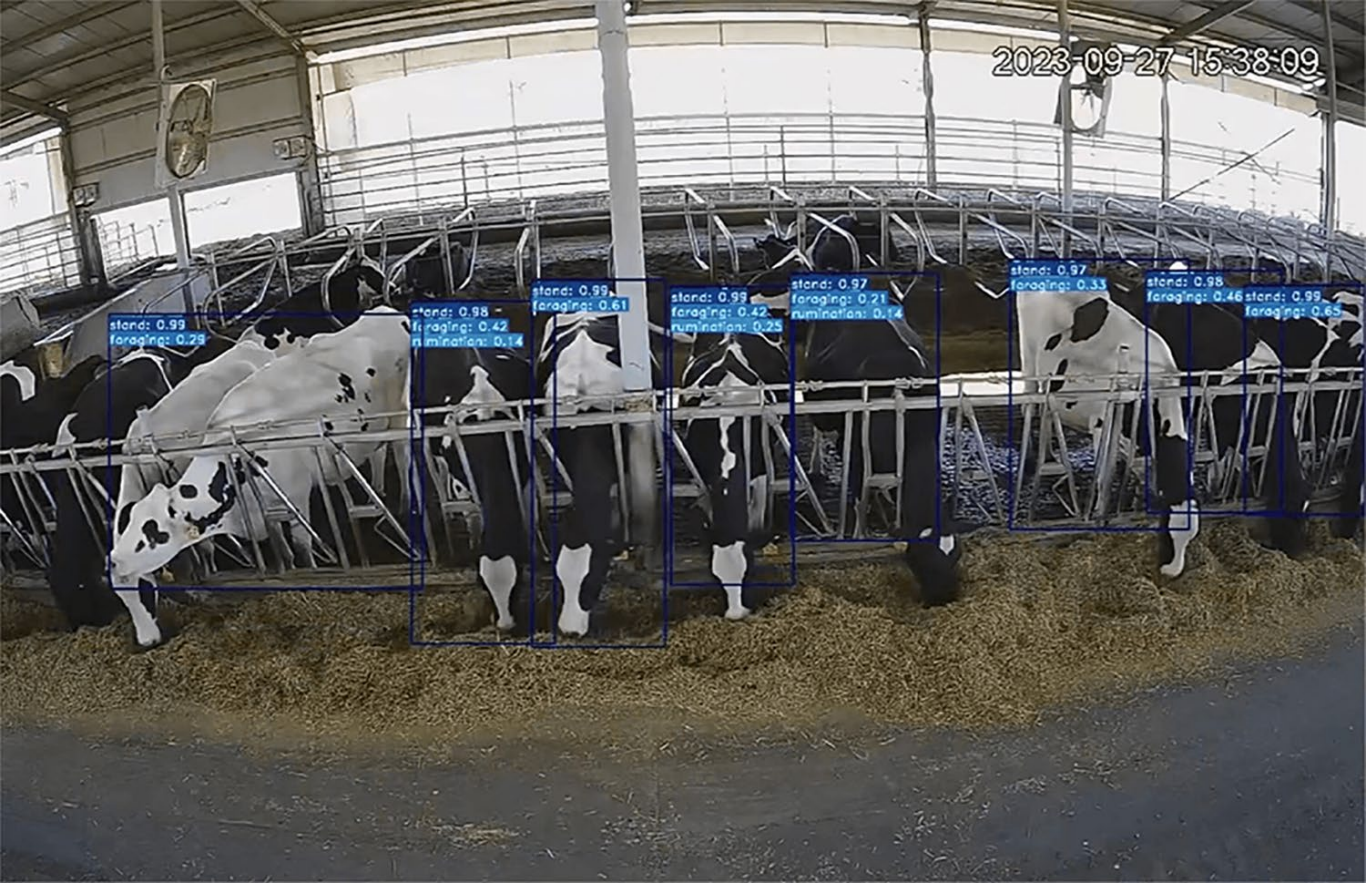
Composite atomic behavior detection results.

Furthermore, we ventured to utilize object detection models in an innovative manner for assessing behavior recognition capabilities. These models were challenged with complex scenarios, encompassing bounding boxes that featured an aggregation of several elementary behaviors, including both posture-related and state behaviors. The resultant findings from these assessments are visually presented in Fig. 9, showcasing the efficacy of our approach.

### Error analysis

For the experimental results of the our baseline model, we give the error analysis on the test set. And the error types are divided into three categories:

- Missed detection behavior: this error typically occurs when rumination behavior and lying down behavior occur simultaneously, and the model fails to capture the subtle variations in rumination behavior during video-level processing.This type of error typically arises from factors like multiple overlapping actions, brief action durations, or actions resembling the background in the video. In experimental results, this error is evident when the model fails to correctly label any actions in certain video frames.
- Missed bounding box behavior: this error occurs when there is a change in the small target box or keyframe annotation box, and the model fails to capture the target due to the small size of the annotation box or the fast movement of the target in the video.This error stems from factors like changes in target size, fast motion, or target occlusion. The model struggles to promptly capture the target’s position and bounding box, leading to inaccuracies in labeling them. In experiments, this error is seen when the model correctly identifies actions in the video but fails to accurately label the target’s position and bounding box.
- False bounding box behavior: this error occurs when the target is similar to the surrounding environment, and the model mistakenly identifies objects in the surrounding environment as the target.This error arises due to factors such as target-background similarity, target complexity, or changes in target posture. The model may misidentify objects in the background as the target, resulting in inaccurate bounding box annotations. In experimental findings, this error manifests when the model annotates the target in the video but with imprecise bounding box positions or misidentifies the target.

**Figure 10:**
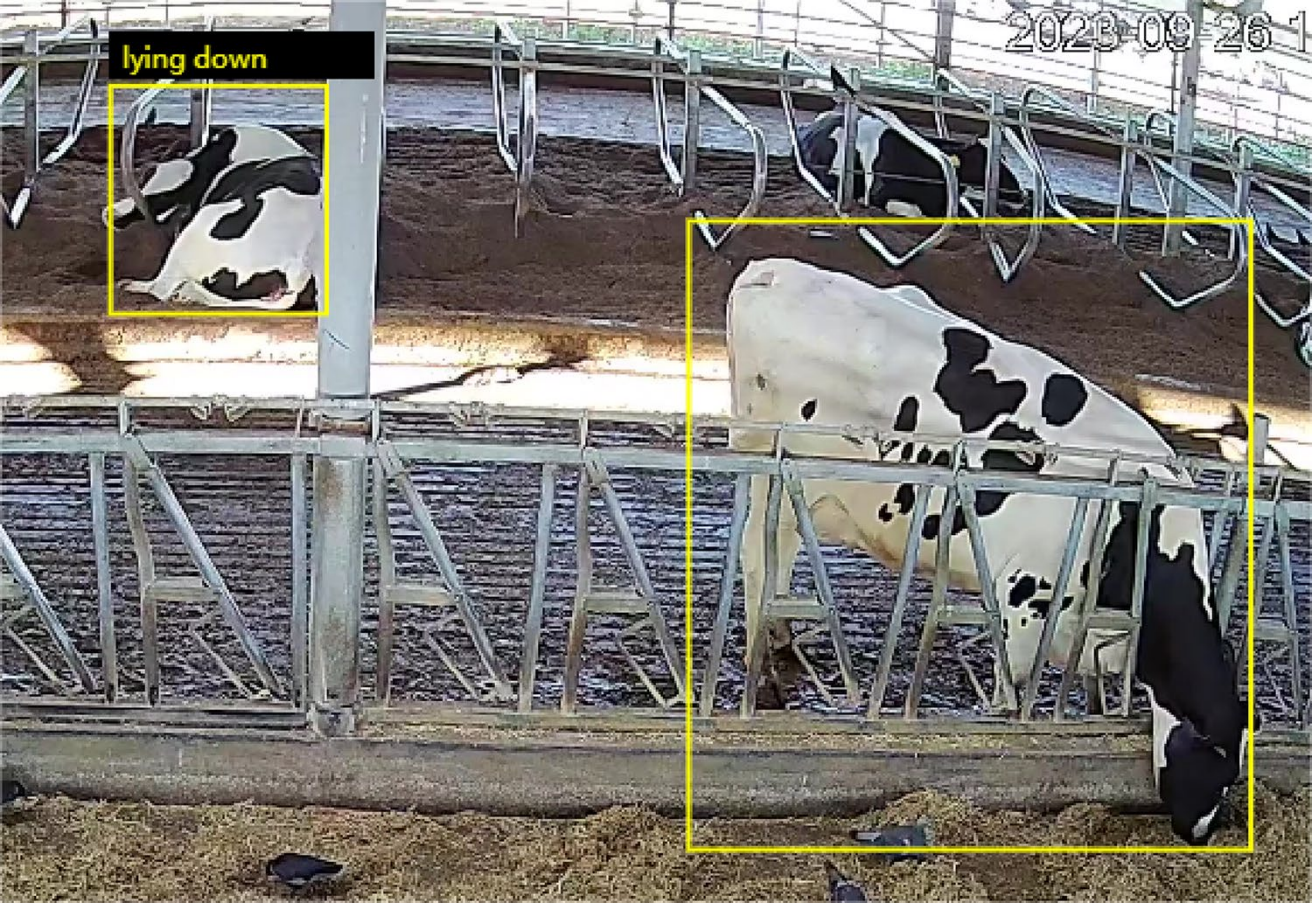
Partial error sample examples.

Taking into account these three types of errors, as depicted in Fig. 10, the cow in the top left corner exhibits an error characterized by partial loss of the target label category. The cow in the bottom left corner, on the other hand, demonstrates an error involving the complete loss of the target label category. Lastly, the cow in the top right corner is associated with an error related to the loss of the target detection box.

When dealing with limited data, one of the primary factors impacting the accuracy of water drinking behavior recognition is the model’s inadequate generalization ability. This limitation results in the model’s real-world performance falling short of expectations, as it struggles to accurately adapt to new scenarios. Another potential issue lies in sample imbalance. In the context of water drinking behavior, certain types of such behavior may occur infrequently, leading to an unequal distribution of samples across different categories in the training data, consequently affecting recognition accuracy.

## Discussion

To address the challenges inherent in recognizing the “drinking” state behavior, future research endeavors are underway to augment the dataset via supplementary sample collection and employment of data augmentation techniques. Specifically, in the fixed drinking zones within pastures, we are reinstalling and readjusting camera strategies to isolate and meticulously capture drinking behaviors. This dedicated effort ensures real-time updates on our Kaggle platform, facilitating vibrant discussions among researchers and thereby promoting a more holistic understanding and learning experience for all stakeholders involved.

Overall, this dataset holds significant value as it ensures that the five behavior classes exhibit variations within reasonable ranges while fine-tuning the model’s hyperparameters. Furthermore, we are committed to continuously updating and optimizing our dataset to support advancements in various related research endeavors.

## Conclusion

In this manuscript, we present the pioneering CBVD-5 dataset, which consolidates a rich repository of five unique cow behavior categories. Comprising 687 meticulously recorded video segments and 206,100 images sourced from a strategic layout of seven cameras across a standardized pasture, this dataset represents a substantial contribution to the field.

To rigorously validate the CBVD-5 dataset’s efficacy, we leveraged transfer learning techniques with the cutting-edge SlowFast model, mirroring the structuring of the esteemed AVA dataset for enhanced cow behavior recognition. Our rigorous experimentation following the dataset’s fine-tuning for this purpose revealed that the model excels not only in accurately discerning various cow behaviors, but also demonstrates sensitivity in detecting minute targets and subtle motion nuances.

We contend that the CBVD-5 dataset constitutes a pivotal benchmark for studies centered around cow behavior recognition, significantly contributing also to advancements in object detection and tracking. This dataset, reflecting meticulous curation and scientific rigor, is made freely accessible to eager scholars upon request, fostering a collaborative ecosystem in advancing our comprehension and management of livestock behaviors for improved welfare and optimized agricultural output.

By providing this invaluable resource, we aim to invigorate global research initiatives, enabling scientists to delve deeper into the intricacies of animal behavior, thereby catalyzing innovations in farming practices and animal welfare science.

## Prospects

Throughout our investigative journey and experimental expansions, we have successfully implemented the YOLO+DeepSort framework for preliminary object detection and real-time tracking of cows. Our upcoming research trajectory aims to refine this focus from an aggregate herd perspective to a more granular, individual-centric approach.

The future plan entails leveraging the YOLO+DeepSort system to assign a unique identifier (ID) to each cow, enabling tailored behavioral recognition centered around these IDs. This individualized strategy is expected to yield more intricate understandings, thereby playing a pivotal role in enhancing the management and health of each dairy cow specifically.

Integrating such an advanced tracking mechanism with intricate behavioral recognition has vast implications for transforming livestock management, facilitating early health issue detection, optimizing feeding schedules, and ultimately improving herd welfare. This underscores the importance of precision livestock farming amidst technological progress. Our ongoing exploration in this vein is poised to set new benchmarks in personalized animal care and significantly contribute to sustainable agricultural practices.

## Ethics statement

The data were gathered from a single location. The staff at that location provided their consent for the use of the data and images in our analysis, dataset, and online publication. This study was assessed by the Laboratory Animal Welfare and Ethics Committee of the Laboratory Animal Center, Inner Mongolia University, and it was confirmed that the study complies with animal protection, animal welfare, and ethical principles, in accordance with national regulations on laboratory animal welfare and ethics, allowing the experiment to proceed.

## Data Availability

All relevant codes included in this study are available upon request by contacting with the corresponding author.And the CBVD-5 dataset is now publicly available at https://www.kaggle.com/datasets/fandaoerji/cbvd-5cow-behavior-video-dataset.
